# Pediatric Tuberculosis in Young Children in India: A Prospective Study

**DOI:** 10.1155/2013/783698

**Published:** 2013-12-10

**Authors:** Sanjay K. Jain, Alvaro Ordonez, Aarti Kinikar, Nikhil Gupte, Madhuri Thakar, Vidya Mave, Jennifer Jubulis, Sujata Dharmshale, Shailaja Desai, Swarupa Hatolkar, Anju Kagal, Ajit Lalvani, Amita Gupta, Renu Bharadwaj

**Affiliations:** ^1^Department of Pediatrics, Johns Hopkins University School of Medicine, 1550 Orleans Street, CRB-II, Room 1.09, Baltimore, MD 21287, USA; ^2^Center for Tuberculosis Research, Johns Hopkins University School of Medicine, 1550 Orleans Street, CRB-II, Room 1.09, Baltimore, MD 21287, USA; ^3^Center for Infection and Inflammation Imaging Research, Johns Hopkins University School of Medicine, 1550 Orleans Street, CRB-II, Room 1.09, Baltimore, MD 21287, USA; ^4^Byramjee Jeejeebhoy Government Medical College, Pune, India; ^5^Department of Medicine, Johns Hopkins University School of Medicine, Baltimore, MD 21287, USA; ^6^National AIDS Research Institute, Pune, India; ^7^Imperial College London, London, UK

## Abstract

*Background*. India has one of the highest tuberculosis (TB) burdens globally. However, few studies have focused on TB in young children, a vulnerable population, where lack of early diagnosis results in poor outcomes. *Methods*. Young children (≤5 years) with suspected TB were prospectively enrolled at a tertiary hospital in Pune, India. Detailed clinical evaluation, HIV testing, mycobacterial cultures, and drug susceptibility testing were performed. *Results*. 223 children with suspected TB were enrolled. The median age was 31 months, 46% were female, 86% had received BCG, 57% were malnourished, and 10% were HIV positive. 12% had TB disease (definite or probable), 35% did not have TB, while TB could not be ruled out in 53%. Extrapulmonary disease was noted in 46%, which was predominantly meningeal. Tuberculin skin test (TST) was positive in 20% of children with TB. Four of 7 (57%) children with culture-confirmed TB harbored drug-resistant (DR) strains of whom 2 (50%) were multi-DR (MDR). In adjusted analyses, HIV infection, positive TST, and exposure to household smoke were found to be significantly associated with children with TB (*P* ≤ 0.04). Mortality (at 1 year) was 3 of 26 (12%) and 1 of 79 (1%), respectively, in children with TB and those without TB (*P* < 0.05). *Conclusions*. Diagnosis of TB is challenging in young children, with high rates of extra-pulmonary and meningeal disease. While the data on DR-TB are limited by the small sample size, they are however concerning, and additional studies are needed to more accurately define the prevalence of DR strains in this vulnerable population.

## 1. Introduction

India has one of the highest tuberculosis (TB) burdens globally, accounting for 20% of the new 8.6 million TB cases annually [1, 2]. While the burden of childhood TB in India is not known, regional data from the World Health Organization (WHO) indicate that sputum microscopy smear-positive TB in children (<14 years old) accounts for 0.6%–3.6% of all reported cases [3]. However, because the majority of children are sputum microscopy smear negative, these data underestimate the true burden of childhood TB. It is estimated that childhood TB constitutes 10–20% of all TB in high-burden countries [4], accounting for 8–20% of TB-related deaths [5–7]. The epidemiology of TB in young children (<5 years old), a vulnerable population where diagnosis and treatment are most challenging, is not well understood, especially in countries with limited public health resources. Furthermore, while several studies have described the pediatric TB epidemic in high HIV/TB coinfection settings such as sub-Saharan Africa [8–10], few studies have focused on TB during early childhood in India. Drug-resistant (DR) strains of *Mycobacterium tuberculosis* are highly prevalent (10–51%) in this region [11, 12], and young children are notoriously difficult to diagnose and treat [13]. In this report, we studied young children in Pune, India, and evaluated the prevalence of both TB and DR-TB. The recognition of TB in vulnerable populations is essential to better define the global TB epidemic and to help direct available healthcare resources.

## 2. Methods

### 2.1. Study Population

Young children (≤5 years) presenting with suspected TB were prospectively enrolled by consecutive sampling from August 15, 2010, to March 28, 2012, at the inpatient and outpatient facilities of the Byramjee Jeejeebhoy Government Medical College (BJGMC; formerly BJMC) and its affiliated private clinics in Pune, India. BJGMC is a large tertiary urban public hospital and the site of an NIH-funded HIV clinical trial unit (CTU). Suspected TB was defined as per the Indian Revised National TB Control Programme (RNTCP) guidelines [14, 15]. This includes children presenting with either fever and/or cough for ≥2 weeks (relaxed from 3 to 2 weeks), with or without weight loss or no weight gain, or showing neurological symptoms like irritability, refusal to feed, headache, vomiting, or altered sensorium and suspected to have TB meningitis. History of contact with a suspected or diagnosed case of TB disease (henceforth TB) within the last 2 years was not used to define a suspect (see Section 4). Exclusion criteria included the inability to obtain informed consent or permission for HIV testing, children on TB treatment or prophylaxis for more than 7 days, and children for whom followup was difficult. Consecutive children meeting the inclusion criteria were enrolled, and written, informed consent for enrolment was obtained from a parent or legal guardian. This study was approved by the Research Ethics Committees of BJGMC (Pune, India) and the Johns Hopkins University (Baltimore, MD).

### 2.2. Study Procedures

History and physical examination were performed on each child by trained study physicians. Weight for age *Z* scores (WAZ) were used to assess the nutritional status (WHO Anthro version 3.2.2, January 2011) with malnutrition defined as WAZ < −2. After appropriate counseling and consent, blood was drawn for HIV testing (positive on two separate HIV ELISA tests or HIV DNA PCR for children <18 months). A standard tuberculin skin test (TST) (5 TU in 0.1 mL; Radiant Parenterals, Ltd., Vaghodia, India) was placed during the initial visit and read at 48–72 hours. Since children were being evaluated for TB, a TST ≥ 5 mm was considered positive [16]. Gastric aspirates (up to 3 consecutive samples) and when indicated other appropriate clinical samples (lymph nodes, cerebrospinal fluid (CSF), pleural fluid, etc.) were obtained for mycobacterial culture. Chest radiographs were also performed on each child and those without highly consistent features of TB were reviewed independently by another radiologist. Computed tomography (CT), magnetic resonance imaging (MRI), and ultrasonography (US) were performed as indicated. All children were evaluated at 2 and 6 weeks after the initial visit and mortality data was collected for up to 1 year.

### 2.3. Microbiology

Clinical samples were processed at BJGMC by trained technicians using standardized protocols. Decontamination was performed with N-acetyl-L-cysteine and sodium hydroxide, and the resuspended pellets were used for acid fast bacilli (AFB) staining and smear microscopy as well as inoculation of Lowenstein Jensen (LJ) slants (one 3 mm loop full of sediment). The remaining ~0.5 mL was cultured using the MGIT 960 TB System (BD, Sparks, MD) and incubated for at least 6 weeks. Positive cultures were confirmed by p-nitrobenzoic acid (PNB) on the MGIT 960. Drug susceptibility testing (DST) for isoniazid (0.1 *μ*g/mL) and rifampin (1.0 *μ*g/mL) was performed using the SIRE kit (BD, Sparks, MD) and confirmed on solid media (proportion method). Genotypic methods, GeneXpert (Cepheid, Sunnyvale, CA; rifampin DST) and INNO-LiPA (Innogenetics NV, Gent, Belgium; isoniazid and rifampin DST), were also utilized.

### 2.4. Data Analysis

Children with suspected TB were classified into the TB diagnostic categories, definite TB (any clinical sample positive for *M. tuberculosis* by culture), probable TB (definition derived from international consensus guidelines [15, 17, 18]), no TB (negative mycobacterial cultures and documented resolution of symptoms without TB treatment), or possible TB (all other children), using an automatic computer algorithm and manually verified independently by the study PI. Noncontinuous variables were reported as frequencies and percentages and compared using Fisher's exact test. Continuous variables were reported as medians and interquartile range (IQR) and compared using a Mann-Whitney nonparametric test. TB was defined as a child with either definite or probable TB and children in the no TB diagnostic category were defined as not having TB. Univariable and multivariable (adjusted) logistic regression analysis was used to estimate unadjusted and adjusted odds ratios and 95% CI. Known risk factors for TB were added to the multivariable model irrespective of their *P* value in the univariable analysis. In addition, a composite variable (exposure to household smoke) comprising the use of wood/biomass fuel for cooking or smoke exposure (smoker(s) in family) was used in the multivariable analysis. All analyses were performed using Excel 2010 (Microsoft, Redmond, WA) or Strata version 11.2 (StataCorp, College Station, TX).

## 3. Results

**Figure 1:**
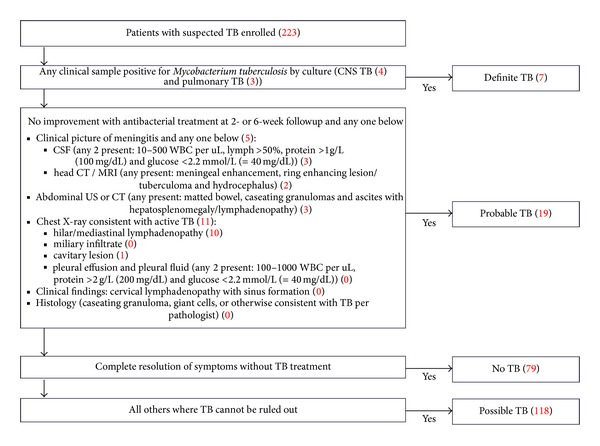
TB diagnostic categories. Criteria used to categorize children with suspected TB into the TB diagnostic categories are outlined. Probable TB definitions are derived from international consensus guidelines [15, 17, 18]. The number of children classified into each diagnostic category is indicated in parentheses. CSF: cerebrospinal fluid; WBC: white blood cells; CT: computer tomography; MRI: magnetic resonance imaging; US: ultrasound.

We screened 309 children with suspected TB and enrolled 223. Eighty six children were excluded: 15 on TB treatment for >7 days; 56 due to inability to followup; 15 due to inability to obtain informed consent (11 due to refusal of the gastric aspirate procedure; one due to transfer to another hospital before consent could be obtained, and three who refused to provide consent for unspecified reasons). The median age of the enrolled children was 31 months (IQR 14 to 44 months), 46% were female, 57% were malnourished, 86% had a BCG scar, and the parent was the primary caregiver for 97%. HIV results were available for 210 of the 223 children, and 21 (10%) were found to be positive. Twenty-six (12%) children were found to have TB (definite or probable), 79 (35%) did not have TB (no TB), while TB could not be ruled out in 118 (53%) and they were classified as possible TB (Figure 1). Characteristics of the enrolled children are listed in Table 1. No significant differences for age, sex, BCG status, and the primary caregiver were noted amongst the different TB diagnostic categories.

For children with TB, 12 of 26 (46%) had extrapulmonary disease, of whom 9 (75%) had TB meningitis. TST was positive in 5 of 25 (20%) of children with TB. Details of the children with definite TB are summarized in Table 2. Of the 7 children, 4 (57%) had extra-pulmonary TB (TB meningitis) and only 1 was HIV positive. DST was initially performed using MGIT and 4 of 7 (57%) children harbored DR strains of whom 2 (50%) were multi-DR (MDR).

### 3.1. Risk Factors Associated with TB

Children with TB were compared with those without TB. No significant differences for age, sex, rates of malnutrition, and BCG status were noted amongst these two groups. Children with TB were more likely to be HIV positive, be TST positive, reside in rural areas, be exposed to biomass cooking fuel, and have a mother with less than primary school education. In adjusted analyses, HIV infection (13.61; 95% confidence interval (CI) 1.44–128.74; *P* = 0.02), positive TST (95.89; 95% CI 7.19–1278.51; *P* < 0.01), and exposure to household smoke (7.43; 95% CI 1.12–49.47; *P* = 0.04) were found to be significantly associated with children with TB. Interestingly, exposure to a known TB case in the past 2 years was not found to be associated with TB.

### 3.2. Initiation of TB Treatment and Mortality

Decision to initiate TB treatment was made independently by the treating physician. Children more likely to have TB (based on the TB diagnostic category) were also more likely to be started on TB treatment within the first 6 weeks (*P* < 0.01) (Table 3). HIV-infected children were more likely to be started on TB treatment (52%) compared with HIV uninfected or unknown patients (*P* = 0.06). Similarly, TST-positive children were also more likely to be started on TB treatment compared to those who were TST negative or unknown (*P* < 0.01). Mortality by 1 year after entry for children with TB was 3 of 26 (12%) and 1 of 79 (1%) among those without TB (*P* < 0.05). There was a nonsignificant trend towards increasing mortality in children more likely to have TB (based on the TB diagnostic category) (*P* = 0.08). Compared with HIV-negative children, HIV-positive children had a higher mortality (*P* < 0.01). However, compared with TST-negative children, those with positive TST did not have a higher mortality (*P* = 1.00). There were no significant differences between children with pulmonary or extra-pulmonary TB in initiation of TB treatment or mortality (*P* ≥ 0.56).

## 4. Discussion

BJGMC serves a poor and underprivileged population and, as expected, the majority of children (57%) were malnourished. As per the cultural practice in this region, the parent was the primary caregiver in the majority (97%). BCG coverage (86%) was similar to the national standard and it was interesting to note that the majority of children with TB (88%) had received prior BCG. While the prevalence of HIV in young children in India is not known, the prevalence nationally in adults is 0.40% and children (<15 years) account for 3.5% of the HIV burden [19, 20]. However, HIV rates are higher in Pune [21, 22], which is a key trucking point in India, and consistent with the higher HIV rates (10%) observed in this study.

We also evaluated the possible risk factors associated with TB. The rates of malnutrition were not different between children with TB compared with those without TB, possibly due to the overall high rates of malnutrition in this cohort. In adjusted analyses, HIV infection, positive TST, and exposure to household smoke were found to be significantly associated with TB. While a positive TST was an independent risk factor for TB, it had low sensitivity (20%) reaffirming that, while a positive TST is helpful, a negative TST does not rule out disease in young children. There are limited data and continued controversy about the association of air pollution and TB risk, and only one prior study has identified exposure to fire-wood smoke as a potential risk factor for TB among children [23]. Therefore, this is a new finding and needs additional followup. Interestingly, exposure to a known TB case in the past 2 years was also not found to be significantly associated with TB. While this may represent an overall high rate of TB in this region, the Revised Pediatric TB guidelines in India have relaxed this requirement [18].

Diagnosis of TB in young children is challenging, as they are less likely to produce adequate specimens for microscopy and culture and more likely to present with extra-pulmonary TB and the paucibacillary nature of childhood TB. Therefore, while culture is not the standard of care in India, we optimized TB detection by utilizing an automated liquid culture media system (MGIT). However, compared with other recently published studies (10.7–16%) [8, 24], the recovery of *M. tuberculosis* was lower (3%) and could be attributed to the high rates of extra-pulmonary TB (46%). While several clinical algorithms and scores for pediatric TB exist, none are sufficiently sensitive or specific. We tried to limit diagnostic bias by applying a rigorous definition for children with probable TB (Figure 1) derived from international consensus guidelines [15, 17, 18]. However, TB could not be definitely ruled out in 53% of TB suspects. It is possible that a longer followup could have made these diagnoses clearer. Interestingly, we found that the reporting of hilar/mediastinal lymphadenopathy, a chest radiography finding consistent with TB, was discordant in 39% of children, when the same study was read by two independent radiologists. Similar discordance in chest radiography performed for the diagnosis of TB in children has been reported previously [25, 26]. While the implications for clinical management of the TB diagnostic categories are unclear, it is reassuring to note that there was a trend correlating mortality by 1 year after entry with the TB diagnostic category, with increasing mortality in children more likely to have TB. Similarly, it is reassuring to note that children more likely to have TB (based on the TB diagnostic category) were also more likely to be started on TB treatment within the first 6 weeks.

Knowledge of region-specific prevalence of DR-TB in young children is extremely important for determining appropriate empiric treatments. A recent meta-analysis by Lew et al. showed that treatment outcomes were substantially worse in patients with DR-TB, which has important implications in resource-limited settings [27]. We therefore performed DST for all culture-confirmed isolates of *M. tuberculosis* which were initially performed using MGIT. While results on subsequent solid media cultures were concordant with MGIT, DST for one isolate (patient 158) was found to be discordant when tested using genotypic methods. This isolate was found to be resistant to both isoniazid and rifampin by phenotypic methods (liquid and solid culture) but susceptible by genotypic methods. While genotypic methods do not detect all resistance conferring mutations, discordance for both rifampin and isoniazid DST is less common [28]. Therefore, laboratory contamination or infection with more than one bacterial strain cannot be entirely ruled out. A recent cross-sectional study by Shah and Chilkar from Mumbai (near Pune) reported that 7% of children (mean age 6.8 years) had DR-TB, of whom 41% was MDR [29]. Interestingly, they reported a much higher rate of DR-TB (65%) in patients with extra-pulmonary disease, consistent with our results, where a large proportion (46%) had extra-pulmonary disease. However, the drug-resistance data should be interpreted with caution, as culture confirmation was successful only in a minority. Furthermore, BJGMC is a tertiary level center, serving a highly underprivileged population, and thus these data may not be fully generalizable nationally. Nonetheless, these data are definitely concerning, and additional studies are needed to more accurately define the prevalence of DR strains in this population.

Finally, it was interesting to note that *M. tuberculosis* strains causing central nervous system (CNS) disease seem to cluster together (see the supplementary figure in the Supplementary Material available online at http://dx.doi.org/10.1155/2013/783698). This is consistent with several clinical reports and animal studies that have observed the association of specific *M. tuberculosis *strains/lineages with TB meningitis [30–33] and suggests that *M. tuberculosis *may possess virulence factors which promote CNS disease [34, 35]. Further analyses of the genetic differences in these strains and detailed analyses of mutations conferring drug resistance are underway.

## 5. Conclusions

Diagnosis of TB is very challenging in young children, and current tools are inadequate. The rates of extra-pulmonary and meningeal TB are very high in this vulnerable population, making the task of definitive diagnosis even more challenging. Furthermore, while the data on DR-TB are limited by the small sample size in the current study, they are however concerning, and additional studies are needed to more accurately define the prevalence of DR in this population.

## Supplementary Material

Supplementary Figure: Cluster dendogram for *M. tuberculosis* isolates from children with definite TB.

## Figures and Tables

**Table 1:** Baseline characteristics of the enrolled children based on TB diagnostic categories. Unless otherwise indicated, the numbers in parentheses indicate percentage.

Characteristics	All, *n* = 223 (100%)	Definite TB, *n* = 7 (3%)	Probable TB, *n* = 19 (9%)	Possible TB, *n* = 118 (53%)	No TB, *n* = 79 (35%)	*P* value
Age (months), median (IQR)	31 (14–44)	12 (6–36)	27 (12–55)	31 (13–43)	31 (19–45)	0.21
Female	102/223 (46)	3/7 (43)	8/19 (42)	55/118 (47)	36/79 (46)	0.99
Parent as primary caregiver	212/219 (97)	7/7 (100)	19/19 (100)	110/115 (96)	76/78 (97)	0.88
BCG scar	192/222 (86)	7/7 (100)	16/19 (84)	100/117 (85)	69/79 (87)	0.87
Malnourished	128/223 (57)	5/7 (71)	9/19 (47)	76/118 (64)	38/79 (48)	0.09
HIV positive	21/210 (10)	1/6 (17)	5/15 (33)	13/112 (12)	2/77 (3)	<0.01
TST positive	27/215 (13)	2/6 (33)	3/19 (16)	20/112 (18)	2/78 (3)	<0.01
Residence						
Urban	65/222 (29)	3/7 (43)	4/19 (21)	31/117 (26)	27/79 (34)	<0.01
Periurban	125/222 (56)	3/7 (43)	8/19 (42)	65/117 (56)	49/79 (62)	
Rural	32/222 (14)	1/7 (14)	7/19 (37)	21/117 (18)	3/79 (4)	
Smoker(s) in family	110/194 (57)	3/7 (43)	7/14 (50)	69/104 (66)	31/69 (45)	0.03
Cooking fuel						
LPG	136/214 (64)	5/7 (71)	6/17 (35)	67/112 (60)	58/78 (74)	<0.01
Kerosene	38/214 (18)	0/7 (0)	3/17 (18)	20/112 (18)	15/78 (19)	
Wood or biomass	40/214 (19)	2/7 (29)	8/17 (47)	25/112 (22)	5/78 (6)	
Mother's education ≤ primary	67/217 (31)	3/7 (43)	7/18 (39)	43/113 (38)	14/79 (18)	0.01
Father's education ≤ primary	67/217 (31)	4/7 (57)	7/18 (39)	37/114 (32)	19/78 (24)	0.19
Exposure to a known TB case in the past 2 years	92/202 (46)	4/7 (57)	3/15 (20)	52/109 (48)	33/71 (46)	0.20

IQR: interquartile range.

**Table 2:** Summary of children with definite TB.

ID	Age in months	Type of disease	HIV status	Disposition at 1 year	MGIT		Solid media		GeneXpert RIF	INNO-LiPA	
					INH	RIF	INH	RIF		INH	RIF
11	48	Meningitis	Negative	Alive	S	S	S	S	S	—	—
21	6	Meningitis	Negative	Alive	S	S	S	S	S	—	—
61	12	Pulmonary	—	Died	R	S	—	—	—	—	—
102	3	Pulmonary	Negative	Alive	S	S	S	S	S	—	—
124	36	Pulmonary	Negative	Alive	R	R	R	R	R	R	R
132	9	Meningitis	Negative	Alive	R	S	R	S	S	R	S
158	36	Meningitis	Positive	Alive	R	R	R	R	S	S	S

INH: isoniazid; RIF: rifampin; S: sensitive; R: resistant.

**Table 3:** Initiation of TB treatment and mortality.

	Treatment initiated within 6 weeks		Mortality within 1 year	
	*n* (%)	*P* value	*n* (%)	*P* value
Type of TB				
Pulmonary TB (*n* = 14)	12 (86%)	0.64	2 (14%)	0.56
Extrapulmonary TB (*n* = 12)	10 (83%)		1 (8%)	
TB diagnostic category				
Definite TB (*n* = 7)	7 (100%)	<0.01	1 (14%)	0.08
Probable TB (*n* = 19)	15 (79%)		2 (11%)	
Possible TB (*n* = 118)	43 (36%)		5 (4%)	
No TB (*n* = 79)	0 (0%)		1 (1%)	
HIV status				
HIV positive (*n* = 21)	11 (52%)	0.06	5 (24%)	<0.01
HIV negative (*n* = 189)	50 (26%)		1 (1%)	
HIV unknown (*n* = 13)	4 (31%)		3 (23%)	
Tuberculin skin test status				
TST positive (*n* = 27)	20 (74%)	<0.01	1 (4%)	0.05
TST negative (*n* = 188)	44 (23%)		6 (3%)	
TST unknown (*n* = 8)	1 (13%)		2 (25%)	
