# Supplementary material for: Pediatric Tuberculosis in Young Children in India: A Prospective Study
Source: Biomed Res Int. 2013 Dec 10;2013:783698. doi: 10.1155/2013/783698 (PMC3872373; doi:10.1155/2013/783698)

Supplementary Figure. Cluster dendogram for *M. tuberculosis* isolates from children with definite TB. Whole genome sequencing (100 bp Paired End) was performed on isolates from 6 of the 7 children with definite TB by Genotypic Technology, Bangalore, India using the Genome Analyzer IIx (Illumina, USA). The sequences for each strain were compared to the reference strain [*M. tuberculosis* H37Rv (NC_000962)]. *M. tuberculosis* strains causing TB meningitis cluster together. This is consistent with several clinical reports and animal studies that have observed the association of specific *M. tuberculosis* strains and lineages with TB meningitis, and suggest that *M. tuberculosis* may possess virulence factors which promote the development of central nervous system disease.

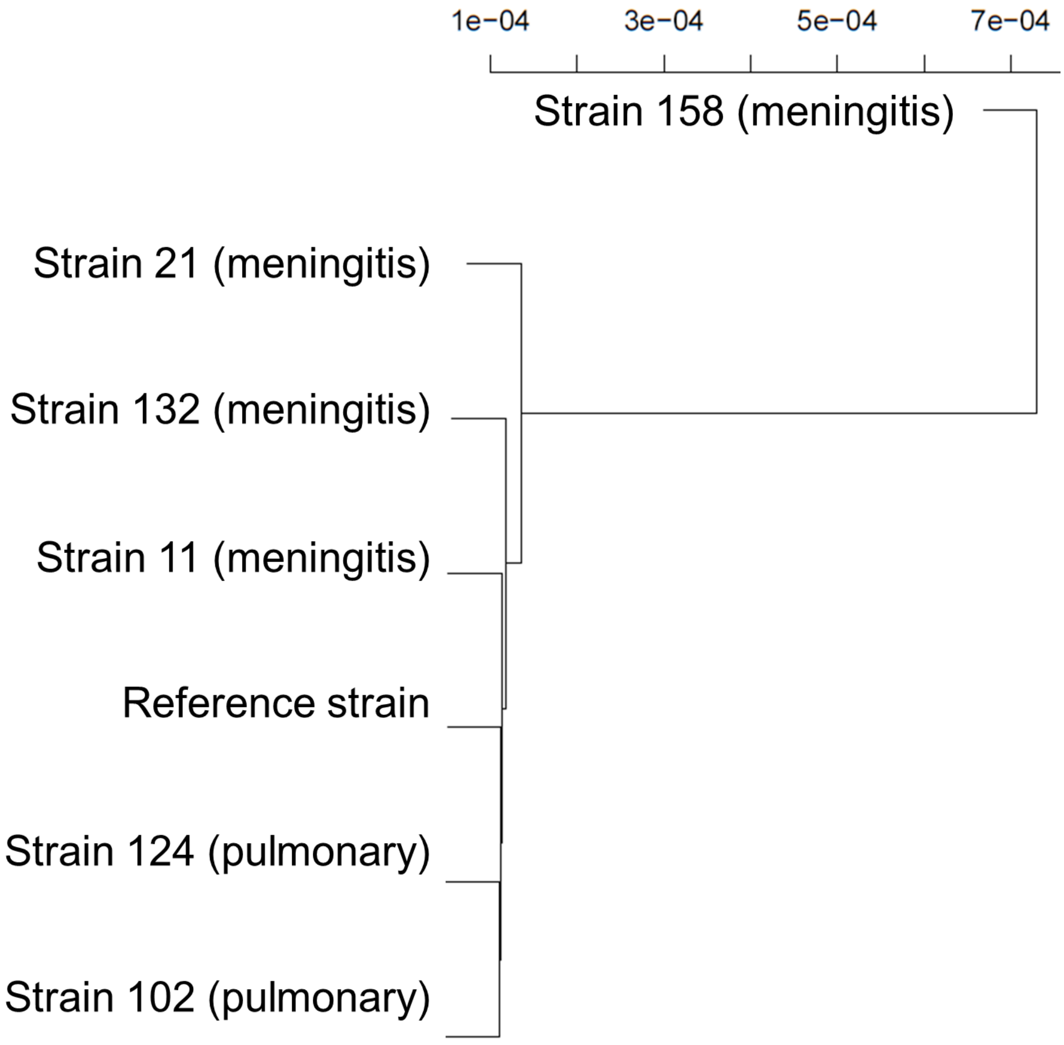

Supplement: Supplementary file 1 — Supplementary Figure: Cluster dendogram for M. tuberculosis isolates from children with definite TB. [file 783698.f1.pdf]
